# The importance of biopsy in clinically diagnosed metastatic lesions in patients with breast cancer

**DOI:** 10.1186/1477-7819-12-93

**Published:** 2014-04-10

**Authors:** Qing Qu, Yu Zong, Xiao-chun Fei, Xiao-song Chen, Cheng Xu, Gu-yin Lou, Kun-wei Shen

**Affiliations:** 1 Department of Oncology, Ruijin Hospital, Shanghai Jiao Tong University School of Medicine, Shanghai 200025, China; 2 Comprehensive Breast Health Center, Ruijin Hospital, Shanghai Jiao Tong University School of Medicine, 197, Ruijin 2 Road, Shanghai 200025, People’s Republic of China; 3 Department of Pathology, Ruijin Hospital, Shanghai Jiao Tong University School of Medicine, Shanghai 200025, China

**Keywords:** Breast cancer, Metastasis, Second malignancy, Biopsy, Receptor status

## Abstract

**Background:**

Receptor status discordance, such as estrogen receptor (ER), progesterone receptor (PR) and human epidermal growth factor receptor 2 (HER2) status between primary breast cancer and metastatic lesions has been reported. The aim of this study was to evaluate the biopsy of clinically diagnosed metastatic lesions and to determine the changes in hormonal receptor and HER2 status of the metastatic lesions.

**Methods:**

Sixty-three patients with clinically diagnosed metastatic breast cancer underwent an excisional biopsy or core needle aspiration guided by computed tomography/ultrasound. ER, PR and HER2 were assessed by immunohistochemistry (IHC).

**Results:**

A total of 48 metastases (76.2%) and nine second primary malignancies (14.3%, seven primary lung cancers and two primary pancreatic cancers) were found. The discrepancies between ER, PR and HER2 status between the primary breast cancer and metastatic lesions were 14.6%, 16.7% and 8.3%, respectively. Six lesions (9.5%) were proved benign upon biopsy.

**Conclusions:**

The biopsy of clinically suspicious metastatic lesions could histologically confirm the diagnosis of metastasis, evaluate discrepancies between ER, PR and HER2 status and exclude secondary malignancy, which might change the therapeutic strategy for breast cancer patients.

## Background

Breast cancer is the most common malignancy affecting women in developed countries. In several cities in China, the incidence has increased dramatically over the past 30 years [1]. Breast cancer patients with early stage disease can be cured, although more than 20% of these patients will eventually develop incurable metastatic disease [2,3]. However, the rates of disease-free survival and the overall survival have increased over the years, largely because adjuvant therapy (chemotherapy, radiation therapy or hormone therapy), has helped prevent local and distant failures. The estrogen receptor (ER), progesterone receptor (PR), and human epidermal growth factor receptor 2 (HER2) are important indicators to determine the prognosis of patients with breast cancer. Breast cancers can be classified into five subtypes according to the ER, PR, HER2 and other markers: luminal A, luminal B, normal breast-like, basal-like, and HER2 overexpressing tumors. ER, PR and HER2 are essential in determining the use of hormone therapy, chemotherapy and targeted therapy [4-7].

Metastasis is the most frequent reason for treatment failure in breast cancer. Diagnosis of metastasis or relapse usually depends on clinical, biological and radiologic evidence [8]. In the metastatic setting, the characteristics of the primary tumors, such as ER, PR and HER2 status, are important to determine the choice of therapeutic strategy. Oncologists often use primary tumor biomarkers to choose endocrine therapy, chemotherapy or targeted therapy for metastatic disease. However, in the last few years, several studies have demonstrated a significant discordance between hormone receptor (HR) status and HER2 status between primary breast cancer and paired asynchronous metastasis [9-13]. If these therapy-predictive markers change throughout tumor progression, then investigating metastatic lesions would provide additional important information about the metastasis, which would enable better management of patients with advanced disease. Due to new therapies, such as targeted therapy and new endocrine therapies, selection of patients using this additional information is crucial for increasing clinical benefit and avoiding unnecessary treatment and toxicities.

The aim of this retrospective study was to investigate the changes in ER, PR and HER2 status in metastatic lesions compared with paired primary breast cancer, and to find changes in the treatment strategies after biopsy confirmation of recurrence with assessment of predictive markers in the patients with suspected breast cancer metastasis.

## Patients and methods

### Patients

A total of 63 patients were diagnosed and treated in the Comprehensive Breast Health Center of Ruijin Hospital, Shanghai Jiaotong University School of Medicine, Shanghai, China between September 2009 and December 2012. Patients who met the following criteria were considered for further analysis: (1) primary breast cancer totally resected, (2) metachronous lesions with suspected metastasis detected by physical examination, ultrasound or computed tomography (CT) without evidence of primary tumor recurrence, (3) multidisciplinary decision for biopsy by surgeons, medical oncologists, radiation therapists, radiologists and pathologists, and (4) complete clinical and follow-up data. Ultimately, 63 patients met the inclusion criteria and were selected for this study. The metastatic histological assessment could be made at metastatic lesion presentation or later after the completion of several lines of treatment. Patients who only underwent cytological investigation of metastasis were excluded from the study.

### Methods

Tissue specimens were obtained by excisional biopsy or core needle aspiration biopsy under guidance of ultrasound or CT. A representative section of the tumor specimen from each case was selected and stained immunohistochemically with a panel of antibodies including ER, PR and HER2. Routine H&E-stained sections were reviewed for the histological tumor type. Immunohistochemistry (IHC) staining was performed on formalin-fixed, paraffin-embedded tissues to evaluate ER, PR and HER2 status. The anti-ER antibody and anti-PR antibody were both from DAKO (Carpinteria, CA, USA). Positive staining for ER/PR was defined as nuclear staining in more than 1% of tumor cells. The anti-HER2 antibody was from Roche (Basel, Switzerland). HER2 was evaluated by an experienced pathologist and scored as 0, 1+, 2+, and 3+ according to the American Society of Clinical Oncology/College of American Pathologists (ASCO/CAP) guidelines. HER2 negativity was considered as HER2 0 or 1+, whereas cases with 2+, or 3+ would be tested using fluorescence *in situ* hybridization (FISH). An amplification ratio HER2/C-17 of > 2 on FISH test was considered as HER2 positivity.

## Results

### Clinical characteristics of patients

Table 1 summarizes the clinical characteristics of the biopsied patients. The median age at breast cancer diagnosis was 53.1 years (range, 30 to 73 years); most patients had invasive ductal histology (98.4%), and most primary tumors were the luminal subtype.

**Table 1:** Characteristics of biopsied patients

**Characteristics**	**n = 63 (%)**
Age (years)	53.1 (30 to 73)
RFS (months)	34.8 (4 to 190)
Tumor size	
T1	32 (50.8%)
T2	27 (42.9%)
T3	4 (6.3%)
Lymph node staging	
0	24 (38.1%)
1	24 (38.1%)
2	9 (14.3%)
3	6 (9.5%)
Histologic type	
IDC	62 (98.4%)
Apocrine carcinoma	1 (1.6%)
Molecule subtype	
Luminal	32 (50.8%)
TNBC	24 (38.1%)
HER2 positive	7 (11.1%)
Adjuvant chemotherapy	
Yes	47 (74.6%)
No	16 (25.4%)
Adjuvant endocrine therapy	
Yes	32 (50.8%)
No	31 (49.2%)
Adjuvant radiotherapy	
Yes	29 (46.0%)
No	34 (54.0%)
Location of suspicious lesions	
Viscera^a^	48 (76.2%)
Soft tissues^b^	15 (23.8%)
Number of suspicious lesions	
Single	11 (17.5%)
Multiple	52 (82.5%)

Abbreviations: IDC, invasive ductal carcinoma, RFS, relapse free survival, TNBC, triple negative breast cancer, ^a^Viscera: liver, pleura, lung, and ovary; ^b^Soft tissues: lymph node and soft tissue.

Table 2 summarizes the biopsy characteristics in these patients. Most patients had undergone biopsy of the lung (38.1%), and the most frequent guidance was using CT (61.9%).

**Table 2:** Characteristics of rebiopsies

**Characteristics**	**n = 63 (%)**
Site of rebiopsy	
Lung	24 (38.1%)
Chest wall	12 (19.0%)
Liver	9 (14.3%)
Lymph nodes	12 (19.0%)
Pancreas	2 (3.2%)
Abdominal wall	1 (1.6%)
Pelvis	1 (1.6%)
Mediastinum	1 (1.6%)
Bone	1(1.6%)
Guide for rebiopsy	
CT	39 (61.9%)
Ultrasound	16 (25.4%)
Incision	8 (12.7%)

### Receptor expression discordance between primary breast cancer and metastatic lesions

**Figure 1:**
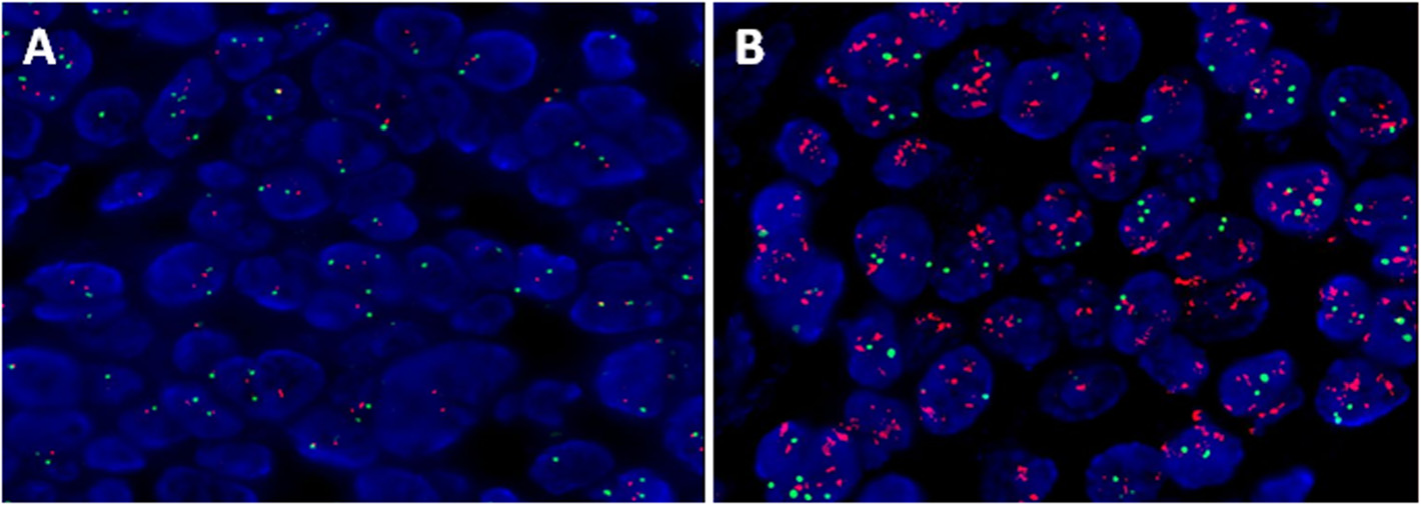
Microscopic findings of HER2 in (A) primary breast tumor (FISH-, magnification x 1,250); (B) liver metastasis (FISH+, magnification x 1,250).

Overall, 48 metastases (76.2%) were confirmed through biopsy. ER and PR status changed between the primary breast tumor and metastatic lesions in 14.6% and 16.7% of patients, respectively (Table 3). There were only four (8.3%) cases that showed a discrepancy in HER2 status (Figure 1). According to the HR and HER2 status of the metastatic lesions, the choice of treatment for the patients was determined at a multidisciplinary treatment meeting. The treatment for the patients without any discrepancy between the primary tumor and metastatic lesions was chosen according to the characteristics of the primary tumors. For the patients with a discrepancy between HR and HER2 status, fourteen patients received modified therapeutic strategies, including ten hormone therapies and four targeted therapies due to a switch in receptors status.

**Table 3:** Change in estrogen receptor and progesterone receptor (ER/PR) and human epidermal growth factor receptor 2 (HER2) status in primary and metastatic tumors

**Primary tumor**	**Metastatic lesions**	**Case number**
ER -	ER +	2
ER +	ER -	5
PR -	PR +	1
PR +	PR -	7
HER2 -	HER2 +	2
HER2 +	HER2 -	2

### Second primary malignancy

**Figure 2:**
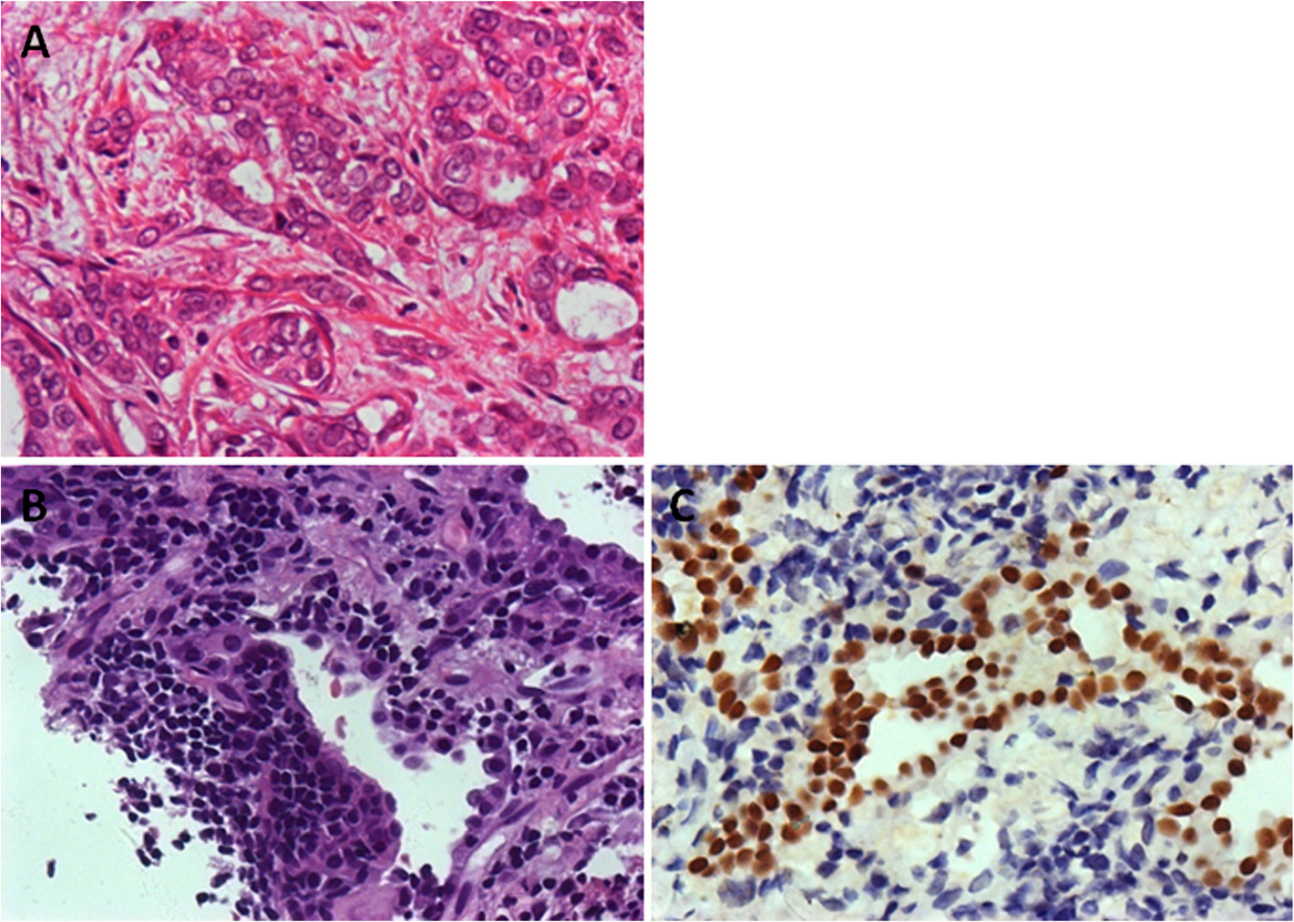
Microscopic findings of (A) primary tumor (magnification x 200), (B) site of lung lesion (magnification x 200); and (C) site of primary lung cancer (IHC TTF-1+, magnification x 200).

In our study, a second primary malignancy was defined as one with a different histological type or different components external to the breast. Nine second primary malignancies (14.3%) were diagnosed upon biopsy, including seven primary lung cancers (Figure 2) and two primary pancreatic cancers.

### Benign lesion

Six patients (9.5%) did not have any evidence of relapse or metastasis upon biopsy. These patients were followed every three months and to date have shown no disease progression.

## Discussion

Breast cancer threatens womens’ health all over the world. Breast cancer is now the second most common malignancy in China [14]. Nearly one half of these patients will eventually develop metastases. In the metastatic setting, the choice of systemic treatment is often based on the biomarker characteristics of the primary tumor, including ER, PR and HER2 status. The breast cancer subtypes defined by ER, PR, and HER2 are helpful to direct treatment and choose endocrine therapy, molecular-targeted therapy and cytotoxic chemotherapy.

Firstly, our study shows that ER, PR, and HER2 are lost and gained in a considerable proportion of patients throughout tumor progression: loss of ER in five cases, gain of ER in two cases, loss of PR in seven cases, gain of PR in one case, loss of HER2 in two cases, and gain of HER2 in two cases. Several studies have also demonstrated the discordance between the primary tumor and metastatic lesions in recent decades [10,13,15,16]. Published studies have found discordance rates for ER status ranging from 10.2 to 56%, PR status ranging from 24.8 to 48.6%, and HER2 status ranging from 2.9 to 16% [15-23]. Jenson’s study has shown that ER status of the metastases changed in 12 to 13% of cases, and HER2 status changed in 5 to 8% [17]. ER/PR and HER2 statuses may be modified by treatment or during disease progression. Liu *et al*. reported that in asynchronous liver metastases, the change in ER, PR and HER2 was 30.4%, 54.3% and 10.9%, respectively. However, in synchronous liver metastases, the change in ER, PR and HER2 were 0, 33.3% and 8.3% respectively [18]. Lindstrom and colleagues demonstrated that one in three patients with breast cancer experience alteration of HR status, and 15% of patients experience a change in HER2 status during tumor progression. In addition, they found that patients with ER-positive primary tumors that changed to ER-negative metastases had an increased mortality risk compared with patients with stable ER-positive tumors. The understanding of tumor dissemination by clarifying the instability of clinically used tumor markers throughout tumor progression could be used to infer both biologic and therapeutic implications in the metastatic setting [19]. The discordance of ER and HER2 in the relapse setting would introduce additional therapeutic choices. The clinical implication of this discordance is important, the loss of HR and HER2 generally indicates resistance to endocrine therapy and trastuzumab, respectively and therefore these patients would benefit from a change of treatment strategy. A total of 14 patients required modification of their treatment strategies in our study, including hormone therapy and targeted therapy because of the altered characteristics in metastatic lesions. Seven patients received endocrine therapy because of the switch of hormone receptor from negative to positive; three patients received chemotherapy because of the negative hormone receptor status in the metastatic lesions which most likely indicates a resistance to endocrine therapy. Two patients were eligible for treatment with trastuzumab because of HER2 status switch to positive; two patients did not use trastuzumab in case of negativity of HER2 in metastatic lesions which probably means a resistance to trastuzumab. Since tumor instability is seen throughout tumor progression, this dynamic highlights the potential need for taking biopsies in a consecutive manner in the advanced setting to optimize clinical decision-making for the patient.

There are several explanations for these alterations. The characteristics of the metastatic lesions could be influenced by factors such as clonal selection by microenvironment and clonal changes induced by adjuvant therapies [24-26]. Additionally, certain factors can influence the IHC results. In the process of IHC staining, many factors could affect results such as fixation time, staining methodology and the size of tissue blocks [27]. Therefore, according to the European Society for Medical Oncology (ESMO) guideline, a biopsy should be performed if there is one single lesion, as well as when the patient has a history of more than one cancer, and when there is suspicion of an alternative diagnosis [28].

Secondly, we found nine second primary malignancies external to the breast through the biopsy of clinically diagnosed metastatic lesions. A number of studies have reported that patients with breast cancer have a high risk of developing a second malignancy, with standardized incidence ratios (SIRs) ranging from 1.15 to 1.6 [29-32]. Brown *et al*. calculated that the SIR for second cancers was 1.15 in patients with a history of breast cancer [29]. Kirova *et al*. reported that the SIR of the primary lung cancer in French patients with a history of the breast cancer is 1.2, but this finding was not significant [30]. Our observation of an increased risk of second primary lung cancer confirms previous findings. Rena *et al*. reported that for a single lung lesion in patients with breast cancer, the rate of primary lung cancer and lung metastasis was 48.1% and 34.2%, respectively [33]. Jensen *et al*. also showed that biopsies of the suspicious metastatic lesions were benign disease or other malignancies in 14% of the patients [17]. In our research, all seven patients with primary lung cancers, confirmed by core needle biopsy, finally had surgery or chemotherapy for the lung cancer. The mechanism involved in the development of second malignancy has not been fully identified. It may be related to the treatment of the breast cancer, such as radiation therapy and anti-estrogen therapy [29,30,34-36].

Thirdly, in our study six patients had a confirmed benign lesion through rebiopsy. The pathologists did not find any evidence of metastasis. These six patients have received regular follow-up every three months. In their study, Rena *et al*. also reported the rate of the benign lesion of single lung lesion after breast surgery was 17.7% [33].

There are several limitations inherent in this study. This analysis was based on a single-center, retrospective study, and therefore we are planning to design prospective studies in the future. In addition, the database spans a short time period (2009 to 2012), during which there has not been enough follow-up data to determine the relationship between the prognosis and the discrepancies of ER, PR and HER2 status.

## Conclusions

In summary, biopsy of suspicious lesions can confirm the relapse of the tumor, exclude the second primary tumor, identify the status of ER, PR and HER2, and allow some patients with breast cancer to benefit from hormone therapy and/or anti-HER2 treatment. The discordance in ER and PR receptor expression between the primary breast tumor and the corresponding metastatic lesions is high, whereas HER2 status remains relatively constant. Therapeutic strategies could be changed after the biopsy because of changes in the tumor characteristics and, therefore, the patients could receive the most effective treatment and avoid unnecessary toxicity. We recommend that biopsy of suspicious metastases and reassessment of ER, PR and HER2 status should become a routine procedure in the treatment of breast cancer patients.

## Abbreviations

CT: computed tomography; ER: estrogen receptor; ESMO: European Society for Medical Oncology; FISH: fluorescence *in situ* hybridization; H&E: hematoxylin and eosin; HR: hormone receptor; HER2: human epidermal growth factor receptor 2; IHC: immunohistochemistry; FISH: Fluorescence in situ hybridization PR, Progesterone receptor; SIRs: standardized incidence ratios.

## Competing interests

The authors have no financial conflicts of interest to declare.

## Authors’ contributions

QQ, X-SC and CX participated in the design of the study and drafted the manuscript. QQ, CX and G-YL participated in data acquisition and analysis. X-CF carried out the pathological immunoassays. QQ, YZ and K-WS carried out the literature review and revised the manuscript. All authors read and approved the final manuscript.
